# A Discussion on the Complications of Pneumonia

**Published:** 1908-01-11

**Authors:** 


### The Hospital

A Journal of

The Medical Sciences and Hospital Administration.

New Series. No. 43, Vol. II. [No. 1115, Vol. XLIII.] SATURDAY, JANUARY 11, 1908.

### A Discussion on the Complications of Pneumonia.

The recent discussion in the medical section of the Royal Society of Medicine is a good illustration of some of the advantages which are likely to attend the amalgamation of the Metropolitan Medical Societies. It is difficult to imagine that any less influential organisation than the one which has now come into existence could have succeeded in obtaining such a body of authoritative information, or could have brought on to one and the same platform so many speakers of high professional rank. A special word of gratitude is due to the Medical Registrars of the various hospitals for their diligent and exact tabulation of facts collected from records covering a long series of years. This laborious work has rendered readily available a mass of information which must be accepted as an exact representation of pneumonia as the disease has occurred over a wide area in this country and when large numbers of cases are brought under review. We will only add the hope that in similar discussions every endeavour will be made to secure the presentation of the experience of those who practise in the provinces, and also of those engaged in general practice. It is a well-known fact that several of the London Societies owe no small part of their success to the interest and assistance of their country members, and though private practitioners may not be able to present so considerable and so exact a collection of statistics as their hospital confrères, they are often able to throw light on various aspects of disease which may have failed to gain attention in hospital practice. The newly formed Society has a mission which is essentially catholic, and the records of its proceedings will be valuable in direct proportion to the area from which it draws its contributors. Especially does this remark apply to such occasions as the one to which reference is here made, that is to the discussion of the various aspects of a disease which is of common incidence and which, to a greater or less extent, enters into the experience of every practitioner.

It may, perhaps, be asked, whether the recent discussion has produced any distinct and direct practical development, or, indeed, whether it has been of value in any respect. There already exist systematic text-books and "systems" which deal with pneumonia on a large and elaborate scale, and these, it may be urged, supply all that is required. Well, first on the question of fact, it may be stated, that no existing text-book presents such a collection of tested and verified clinical and pathological observations as is to be found in the hospital records to which allusion has already been made. And yet again, a discussion allows, or even encourages, a profession of personal views and impressions and the suggestion of degrees of confidence or uncertainty in regard to particular points which can hardly be admitted in a systematic treatise. It is indeed something like a professional consultation on a large scale, and both those who take part in it and those who study its record acquire an appreciation of values as well as a knowledge of individual occurrences and facts. That it is the function of a medical society to offer an opportunity for the announcement of new developments and for the study of rare and exceptional diseases no one denies. But it is not less important that the experiences of many observers in connection with comparatively common clinical conditions should be occasionally presented in such a fashion that they can be compared and tested in debate, can be summarised for the benefit of the profession generally, and can be utilised as a starting point for fresh advances. Such an arrangement has many advantages, and not the least of these are the detection of weak places in our theoretical and practical position and the critical scrutiny of ancient beliefs in the light of modern knowledge. Thus, so it seems to us, the Royal Society of Medicine showed a sound recognition of its responsibilities when it determined to devote one of its earliest meetings to a discussion on so frequent and well-recognised a disease as pneumonia.

On the numerous details of the discussion it is, of course, impossible for us here to enter. But there are one or two points to which a general allusion may be made. First may be noted the satisfactory fact that even in hospital cases complications of any kind in pneumonia are uncommon. In an analysis of 7,868 cases there were only 3.7 per cent. of cases of empyema, and this was by far the most common complication of the disease. Yet, putting complications on one side, pneumonia is shown to carry a serious mortality, for in the above total of cases there were no fewer than 1,722 deaths, that is 21.8 per cent. The figures also support the generally received opinion that the fatal influence of the disease increases with each decade, as if, for some reason or other, the bodily resistance to the pneumococcus gradually declined as age advances. At the same time, it is quite true, as Sir Douglas Powell has recently pointed out, that very old people do, not very infrequently, make a perfectly good recovery from an attack of pneumonia. Another point of interest is the much greater frequency of the disease in the male sex. Doubtless this is partly due to the greater exposure entailed by the occupations in which men are engaged; but it is also probable, as suggested by Dr. Hector Mackenzie, that the habit of spitting, by men, gives opportunity for the spreading of the pneumococcal infection to their fellows. The recital of such complications as arthritis, meningitis, otitis, etc., emphasises the change of view which removes pneumonia from the category of diseases of the lung and places it among the general infections. Two practical questions arise from this. The one, what degree of care should be taken to prevent the pneumonic patient proving a danger to his friends. And the second: Is anything to be expected from serum treatment? Regarding the latter it may at once be said that, whatever the future may bring, nothing so far has been actually accomplished. In reference to direct infection, the risk cannot be said to be great; but it exists, and such measures as the use of mouth washes and the disinfection of the sputa ought to be practised.

